# Well-Posedness of Nonlocal Parabolic Differential Problems with Dependent Operators

**DOI:** 10.1155/2014/519814

**Published:** 2014-01-12

**Authors:** Allaberen Ashyralyev, Asker Hanalyev

**Affiliations:** ^1^Department of Mathematics, Fatih University, 34500 Istanbul, Turkey; ^2^ITTU, 74400 Ashgabat, Turkmenistan; ^3^Tejen High School, Tejen, Turkmenistan; ^4^Department of Mathematics, Turkmen State University, 74400 Ashgabat, Turkmenistan

## Abstract

The nonlocal boundary value problem for the parabolic differential equation *v*'(*t*) + *A*(*t*)*v*(*t*) = *f*(*t*)  (0 ≤ *t* ≤ *T*), *v*(0) = *v*(*λ*) + *φ*, 0 < *λ* ≤ *T* in an arbitrary Banach space *E* with the dependent linear positive operator *A*(*t*) is investigated. The well-posedness of this problem is established in Banach spaces *C*_0_^*β*,*γ*^(*E*_*α*−*β*_) of all *E*_*α*−*β*_-valued continuous functions *φ*(*t*) on [0, *T*] satisfying a Hölder condition with a weight (*t* + *τ*)^*γ*^. New Schauder type exact estimates in Hölder norms for the solution of two nonlocal boundary value problems for parabolic equations with dependent coefficients are established.

## 1. Introduction: A Cauchy Problem

It is known that (see, e.g., [1–5] and the references given therein) several applied problems in fluid mechanics, physics, and mathematical biology were formulated into nonlocal mathematical models. In general, such nonlocal problems were not well studied.

Before going to discuss the well-posedness of nonlocal boundary value problem, we will give the definition of positive operators in a Banach space and introduce the fractional spaces generated by positive operators that will be needed in the sequel.

Let *E* be a Banach space and let *A* : *D*(*A*)⊂*E*→*E* be a linear unbounded operator densely defined in *E*. The operator *A* is said to be positive in the Banach space *E* if its spectrum *σ*_*A*_ lies in the interior of the sector of angle *ϕ*, 0 < 2*ϕ* < 2*π*, symmetric with respect to the real axis, and if, on the edges of this sector, *S*_1_(*ϕ*) = {*ρe* ^*iϕ*^ : 0 ≤ *ρ* ≤ *∞*} and *S*_2_(*ϕ*) = {*ρe* ^−*iϕ*^ : 0 ≤ *ρ* ≤ *∞*}, and outside of the sector the resolvent (*λ*−*A*)^−1^ is subject to the bound

(1) $$\begin{array}{c} {||{\left(A-\lambda \right)}^{-1}||}_{E\to E}\leq \frac{M}{1+\left|\lambda \right|}. \end{array}$$

The infimum of all such angles *ϕ* is called the spectral angle of the positive operator *A* and is denoted by *φ*(*A*, *E*). We call *A* strongly positive in the Banach space *E* if its spectral angle *φ*(*A*, *E*) < *π*/2.

For positive operator *A* in the Banach space *E*, let us introduce the fractional spaces *E*_*β*_ = *E*_*β*_(*E*, *A*) (0 < *β* < 1) consisting of those *ν* ∈ *E* for which the norm

(2) $$\begin{array}{c} {||\nu ||}_{E_{\beta }}=\underset{\lambda >0}{\sup}\lambda^{\beta }{||A{\left(\lambda +A\right)}^{-1}\nu ||}_{E}+{||\nu ||}_{E} \end{array}$$

is finite.

In the paper [6], the well-posedness in spaces of smooth functions of the nonlocal boundary value problem

(3) $$\begin{array}{c} v^{\prime }\left(t\right)+Av\left(t\right)=f\left(t\right)\;\left(0\leq t\leq T\right),\\ v\left(0\right)=v\left(\lambda \right)+\mu \;\left(0<\lambda \leq T\right) \end{array}$$

for the differential equation in an arbitrary Banach space *E* with the strongly positive operator *A* was established. The importance of coercive inequalities (well-posedness) is well known [7, 8].

Noted that theory and methods for approximate solutions of local and nonlocal boundary value problems for evolution differential equations have been studied extensively by many researchers (see [9–39] and the references therein).

Before going to establish theorems on the well-posedness of nonlocal boundary value problem for parabolic equations in an arbitrary Banach space with the strongly positive dependent space operators, let us consider the abstract Cauchy problem for the differential equation

(4) $$\begin{array}{c} v^{\prime }\left(t\right)+A\left(t\right)v\left(t\right)=f\left(t\right)\;\left(0\leq t\leq T\right),\;\;v\left(0\right)=v_{0} \end{array}$$

in an arbitrary Banach space *E* with the strongly positive operators *A*(*t*) in *E* with domain *D*(*A*(*t*)) = *D*, independent of *t* and dense in *E*.

A function *v*(*t*) is called a solution of the problem (4) if the following conditions are satisfied.

- *v*(*t*) is continuously differentiable on the segment  [0, *T*]. The derivative at the endpoints of the segment is understood as the appropriate unilateral derivatives.
- The element *v*(*t*) belongs to *D* = *D*(*A*(*t*)) for all *t* ∈ [0, *T*], and the function *A*(*t*)*v*(*t*) is continuous on [0, *T*].
- *v*(*t*) satisfies the equation and the initial condition (4).

A solution of problem (4) defined in this manner will from now on be referred to as a solution of problem (4) in the space *C*(*E*) = *C*([0, *T*], *E*). Here, *C*(*E*) stands for the Banach space of all continuous functions *φ*(*t*) defined on [0, *T*] with values in *E* equipped with the norm

(5) $$\begin{array}{c} {||\varphi ||}_{C(E)}=\underset{0\leq t\leq T}{\max}{||\varphi \left(t\right)||}_{E}. \end{array}$$

From the existence of the such solutions, it evidently follows that *f*(*t*) ∈ *C*(*E*) and *v*_0_ ∈ *D*.

We say that the problem (4) is well posed in *C*(*E*) if the following conditions are satisfied.

(1) Problem (4) is uniquely solvable for any *f*(*t*) ∈ *C*(*E*) and any *v*_0_ ∈ *D*. This means that an additive and homogeneous operator *v*(*t*) = *v*(*t*; *f*(*t*), *v*_0_) is defined which acts from *C*(*E*) × *D* to *C*(*E*) and gives the solution of problem (4) in *C*(*E*).
(2) *v*(*t*; *f*(*t*), *v*_0_), regarded as an operator from *C*(*E*) × *D* to *C*(*E*), is continuous. Here, *C*(*E*) × *D* is understood as the normed space of the pairs (*f*(*t*), *v*_0_), *f*(*t*) ∈ *C*(*E*), and *v*_0_ ∈ *D*, equipped with the norm
  (6) $$\begin{array}{c} {||\left(f\left(t\right),v_{0}\right)||}_{C(E)\times D}={||f||}_{C(E)}+{||v_{0}||}_{D}. \end{array}$$

By Banach's theorem, in *C*(*E*) and these properties, one has coercive inequality

(7) $$\begin{array}{c} {||v^{\prime }||}_{C(E)}+{||A(\cdot )v||}_{C(E)}\leq M_{C}\left[{||f||}_{C(E)}+{||v_{0}||}_{D}\right], \end{array}$$

where *M*_*C*_  (1 ≤ *M*_*C*_ < +*∞*) does not depend on  *v*_0_  and *f*(*t*).

Inequality (7) is called the coercivity inequality in *C*(*E*) for (4). If *A*(*t*) = *A*, then the coercivity inequality implies the analyticity of the semigroup exp{−*sA*}  (*s* ≥ 0), that is, the following estimates

(8) $$\begin{array}{c} {||\exp\left(-sA\right)||}_{E\to E},{||sA\exp\left(-sA\right)||}_{E\to E}\leq M\left(s>0\right) \end{array}$$

hold for some *M* ∈ [1, +*∞*). Thus, the analyticity of the semigroup exp{−*sA*}  (*s* ≥ 0) is  necessary for the well-posedness of problem (4) in *C*(*E*). Unfortunately, the analyticity of the semigroup exp{−*sA*}  (*s* ≥ 0) is not sufficient for the well-posedness of problem (4) in *C*(*E*).

Suppose that, for each *t* ∈ [0, *T*], the operator −*A*(*t*) generates an analytic semigroup exp{−*sA*(*t*)}  (*s* ≥ 0) with exponentially decreasing norm, when *s* → +*∞*, that is, the following estimates

(9) ||exp(−sA(t))||E→E,||sA(t)exp(−sA(t))||E→E  ≤Me−δs(s>0)

hold for some *M* ∈ [1, +*∞*),  *δ* ∈ (0, +*∞*). From this inequality, it follows that the operator *A* ^−1^(*t*) exists and is bounded, and hence *A*(*t*) is closed in *C*(*E*).

Suppose that the operator *A*(*t*)*A* ^−1^(*s*) is Hölder continuous in *t* in the uniform operator topology for each fixed *s*, that is,

(10) $$\begin{array}{c} {||\left[A\left(t\right)-A\left(\tau \right)\right]A^{-1}\left(s\right)||}_{E\mu \to E\mu }\leq M{\left|t-\tau \right|}^{\varepsilon },\;0<\varepsilon \leq 1, \end{array}$$

where *M* and *ε* are positive constants independent of *t*,  *s*, and *τ* for 0 ≤ *t*, *s*, *τ* ≤ *T*,  0 ≤ *μ* ≤ *ε*.

From (9) and (10), it follows that *E*_*α*_(*E*, *A*(*t*)) = *E*_*α*_(*E*, *A*(0)) for all 0 < *α* < 1  and 0 ≤ *t* ≤ *T* (see, [14]).

An operator-valued function *v*(*t*, *s*), defined and strongly continuous jointly in *t* and *s* for 0 ≤ *s* < *t* ≤ *T*, is called a fundamental solution of (4) if

(1) the operator *v*(*t*, *s*) is strongly continuous in *t* and *s* for 0 ≤ *s* < *t* ≤ *T*,
(2) the following identity holds:
  (11) v(t,s)=v(t,τ)v(τ,s),v(t,t)=Ifor  0≤s≤τ≤t≤T,
(3) the operator *v*(*t*, *s*) maps the region *D* into itself; the operator *u*(*t*, *s*) = *A*(*t*)*v*(*t*, *s*)*A* ^−1^(*s*) is bounded and strongly continuous in *t* and *s* for 0 ≤ *s* < *t* ≤ *T*,
(4) on the region *D*, the operator *v*(*t*, *s*) is strongly differentiable relative to *t* and *s*, while
  (12) ∂v(t,s)∂t=−A(t)v(t,s),∂v(t,s)∂s=v(t,s)A(s).

*v*(*t*, *s*) is also called development family, evolution operator, Green's function, and so forth [14].

If the function *f*(*t*) is not only continuous, but also continuously differentiable on [0, *T*], and *v*_0_ ∈ *D*, it is easy to show that the formula

(13) $$\begin{array}{c} v\left(t\right)=v\left(t,0\right)v_{0}+\overset{t}{\underset{0}{\int }}v\left(t,s\right)f\left(s\right)ds \end{array}$$

gives a solution of problem (4).

Now, we will give lemmas and estimates from [14] concerning the semigroup exp{−*sA*(*t*)}  (*s* ≥ 0) and the fundamental solution *v*(*t*, *s*) of (4) and theorem on well-posedness of (4) which will be useful in the sequel.

**Lemma 1**

For any 0 < *s* < *s* + *τ* < *T*,  0 ≤ *t* ≤ *T*, and 0 ≤ *α* ≤ 1, one has the inequality

(14) ||exp(−sA(t))−exp(−(s+τ)A(t))||E→E  ≤Mτα(s+τ)α,

where *M* does not depend on *α*, *t*, *s*, and *τ*.

**Lemma 2**

For any 0 ≤ *s*,   *τ*,  *t* ≤ *T* and 0 ≤ *ε* ≤ 1, the following estimates hold:

(15) ||[exp(−tA(τ))−exp(−sA(τ))]A−1(τ)||E→E  ≤M|t−s|εe−δmin{t,s},||A(t)[exp(−tA(τ))−exp(−sA(τ))]A−2(τ)||E→E  ≤M|t−s|εe−δmin{t,s},

where *M*≥0 and *δ* > 0 do not depend on *ε*, *t*, *s*, and *τ*.

**Lemma 3**

For any 0 ≤ *s*<*t* ≤ *T* and *u* ∈ *D*, the following identities hold:

(16) v(t,s)u=exp{−(t−s)A(s)}u+∫stv(t,z)[A(s)−A(z)]A−1(s) ×exp{−(z−s)A(s)}A(s)u dz,

(17) v(t,s)u=exp{−(t−s)A(t)}u+∫stexp{−(t−z)A(t)} ×[A(z)−A(t)]v(z,s)u dz.

**Lemma 4**

For any 0 ≤ *s*<*t* ≤ *t* + *r* ≤ *T*, the following estimates hold:

(18) ||v(t,s)||E→E≤M,

(19) ||A(t)v(t,s)A−1(s)||E→E≤M,

(20) ||A(t)v(t,s)||E→E≤Mt−s,

where *M*≥0 does not depend on *t* and *s*.

A function *v*(*t*) is said to be a solution of problem (4) in *F*(*E*) if it is a solution of this problem in *C*(*E*) and the functions  *v*′(*t*) and *A*(*t*)*v*(*t*) belong to *F*(*E*).

As in the case of the space *C*(*E*), we say that the problem (4) is well posed in *F*(*E*) if the following two conditions are satisfied.

(1) For any *f* ∈ *F*(*E*) and *v*_0_ ∈ *D*, there exists the unique solution *v*(*t*) = *v*(*t*; *f*(*t*), *v*_0_) in *F*(*E*) of problem (4). This means that an additive and homogeneous operator *v*(*t*; *f*(*t*), *v*_0_) is defined which acts from *F*(*E*) × *D* to *F*(*E*) and gives the solution of (4) in *F*(*E*).
(2) *v*(*t*; *f*(*t*), *v*_0_), regarded as an operator from *F*(*E*) × *D* to *F*(*E*), is continuous. Here, *F*(*E*) × *D* is understood as the normed space of the pairs (*f*(*t*), *v*_0_),*f*(*t*) ∈ *F*(*E*), and *v*_0_ ∈ *D*, equipped with the norm
  (21) $$\begin{array}{c} {||\left(f\left(t\right),v_{0}\right)||}_{F(E)\times D}={||f||}_{F(E)}+{||v_{0}||}_{D}. \end{array}$$

We set *F*(*E*) equal to *C*_0_^*β*,*γ*^(*E*), (0 ≤ *γ* ≤ *β*, 0 < *β* < 1) space, obtained by completion of the set of all smooth *E*-valued functions *φ*(*t*) on [0, *T*] with respect to the norm

(22) ||φ||C0β,γ(E)=max0≤t≤T||φ(t)||E+sup0≤t<t+τ≤T(t+τ)γ||φ(t+τ)−φ(t)||Eτβ.

Let us give the following theorems on well-posedness of (4) in *C*_0_^*β*,*γ*^(*E*_*α*−*β*_), 0 ≤ *γ* ≤ *β* ≤ *α*, 0 < *α* < 1 from [14]. To these, there correspond the spaces of traces *E*_*α*−*β*_^*β*,*γ*^, which consist of elements *w* ∈ *E* for which the norm

(23) |w|α−ββ,γ=max0≤z≤T||e−zA(t)w||Eα−β+sup0≤z<z+τ≤Tτ−β(z+τ)γ||[e−(z+τ)A(t)−e−zA(t)]w||Eα−β

is finite.

**Theorem 5**

Suppose *v*_0_′ ∈ *E*_*α*−*γ*_,  *f*(*t*) ∈ *C*_0_^*β*,*γ*^(*E*_*α*−*β*_)  (0 ≤ *γ* ≤ *β* ≤ *α*, 0 < *α* < 1). Suppose that the assumptions (9) and (10) hold and 0 < *α* ≤ *ε* < 1. Then, for the solution *v*(*t*) in *C*_0_^*β*,*γ*^(*E*_*α*−*β*_) of the Cauchy problem (4), the coercive inequality

(24) ||v′||C0β,γ(Eα−β)+||A(·)v||C0β,γ(Eα−β)+||v′||C(Eα−γ)  ≤Mα−1(1−α)−1[||v0′||Eα−γ+||f||C0β,γ(Eα−β)]

holds, where *M* does not depend on *α*, *β*, *γ*, *v*_0_′, and *f*(*t*).

**Theorem 6**

Suppose *v*_0_′ ∈ *E*_*α*−*β*_^*β*,*γ*^,  *f*(*t*) ∈ *C*_0_^*β*,*γ*^(*E*_*α*−*β*_)  (0 ≤ *γ* ≤ *β* ≤ *α*, 0 < *α* < 1). Suppose that the assumptions (9) and (10) hold and 0 < *α* ≤ *ε* < 1. Then, for the solution *v*(*t*) in *C*_0_^*β*,*γ*^(*E*_*α*−*β*_) of the Cauchy problem (4), the coercive inequality

(25) ||v′||C0β,γ(Eα−β)+||A(·)v||C0β,γ(Eα−β)+||v′||C(Eα−ββ,γ)  ≤M[|v0′|α−ββ,γ+α−1(1−α)−1||f||C0β,γ(Eα−β)]

holds, where *M* does not depend on *α*, *β*, *γ*, *v*_0_′, and *f*(*t*).

Note that the spaces of smooth functions *C*_0_^*β*,*γ*^(*E*_*α*−*β*_)  (0 ≤ *γ* ≤ *β* ≤ *α*, 0 < *α* < 1), in which coercive solvability has been established, depend on the parameters *α*, *β*, and *γ*. However, the constants in the coercive inequalities depend only on *α*. Hence, we can choose the parameters *β* and *γ* freely, which increases the number of functional spaces in which problem (4)  is well posed. In particular, Theorems 5 and 6 imply theorems on well-posedness of the nonlocal boundary value problem (4) in *C*(*E*_*α*_)  (0 < *α* < 1).

Finally, in the paper [40], the initial-value problem

(26) $$\begin{array}{c} \frac{dv\left(t\right)}{dt}+D_{t}^{1/2}v\left(t\right)+A\left(t\right)v\left(t\right)=f\left(t\right),\;0<t<1,v\left(0\right)=0 \end{array}$$

for the fractional parabolic equation in an arbitrary Banach space *E* with the strongly positive operators *A*(*t*) was investigated. Here, *D*_*t*_^1/2^ = *D*_0+_^1/2^ is standard Riemann-Liouville's derivative of order 1/2. The well-posedness of problem (26) in *C*(*E*_*α*_) spaces was established. New exact estimates in Hölder norms for the solution of initial-boundary value problems for fractional parabolic equations were obtained.

In the paper [41], the nonlocal boundary value problem for the parabolic differential equation

(27) $$\begin{array}{c} v^{\prime }\left(t\right)+A\left(t\right)v\left(t\right)=f\left(t\right)\;\left(0\leq t\leq T\right),\\ v\left(0\right)=v\left(\lambda \right)+\varphi ,\;0<\lambda \leq T \end{array}$$

in an arbitrary Banach space *E* with the strongly positive operators *A*(*t*) in *E* with domain *D*(*A*(*t*)) = *D*, independent of *t* and dense in *E*, was investigated. The well-posedness of problem (27) in *C*_0_^*β*,*γ*^(*E*) spaces was established. New exact estimates in Hölder norms for the solution of three nonlocal boundary value problems for parabolic equations were obtained.

In the present paper, the well-posedness of problem (27) in *C*_0_^*β*,*γ*^(*E*_*α*−*β*_) spaces is established. New Schauder type exact estimates in Hölder norms for the solution of two nonlocal boundary value problems for parabolic equations with dependent coefficients are established.

The paper is organized as follows.  Section 1 is introduction. In Section 2, new theorems on well-posedness of problem (27) in *C*_0_^*β*,*γ*^(*E*_*α*−*β*_) spaces are established. In Section 3, theorems on the coercive stability estimates for the solution of two nonlocal boundary value parabolic problems are obtained. Finally, Section 4 is conclusion.

## 2. Well-Posedness of Nonlocal Boundary Value Problem (27)

Now, we will give lemmas on the fundamental solution *v*(*t*, *s*) of (4) from paper [41].

**Lemma 7**

Assume that *A*(*t*)*A*(*p*)^−1^ = *A*(*t* + *λ*)*A*(*p*)^−1^, *p* ∈ [0, *T*] for any 0 ≤ *t* ≤ *t* + *λ*. Then, for any 0 ≤ *s* < *t* ≤ *t* + *λ* and *u* ∈ *D*, the following identity holds

(28) $$\begin{array}{c} v\left(t,s\right)u=v\left(t+\lambda ,s+\lambda \right)u. \end{array}$$

**Lemma 8**

Under the assumption of Lemma 7 there exists the inverse of the operator *I* − *v*(*λ*, 0) in *E* and the following estimate holds:

(29) ||(I−v(λ,0))−1||E→E≤M(λ),

(30) ||A(0)(I−v(λ,0))−1A(λ)−1||E→E≤M(λ).

A function *v*(*t*) is called a solution of the problem (27) if the following conditions are satisfied.

- *v*(*t*) is continuously differentiable on the segment [0, *T*].
- The element *v*(*t*) belongs to *D* for all *t* ∈ [0, *T*], and the function *A*(*t*)*v*(*t*) is continuous on [0, *T*].
- *v*(*t*) satisfies the equation and the nonlocal boundary condition (27).

We say that the problem (27) is well posed in *C*(*E*) if the following conditions are satisfied.

(1) Problem (27) is uniquely solvable for any *f*(*t*) ∈ *C*(*E*) and any *φ* ∈ *D*. This means that an additive and homogeneous operator *v*(*t*) = *v*(*t*; *f*(*t*), *φ*) is does not depend on defined which acts from *C*(*E*) × *D* to *C*(*E*) and gives the solution of problem (4) in *C*(*E*).
(2) *v*(*t*; *f*(*t*), *φ*), regarded as an operator from *C*(*E*) × *D* to *C*(*E*), is continuous. Here, *C*(*E*) × *D* is understood as the normed space of the pairs (*f*(*t*), *φ*),  *f*(*t*) ∈ *C*(*E*), and *φ* ∈ *D*, equipped with the norm
  (31) ||(f(t),φ)||C(E)×D=||f||C(E)+||φ||D.

By Banach's theorem in *C*(*E*) and these properties, one has coercive inequality

(32) $$\begin{array}{c} {||v^{\prime }||}_{C(E)}+{||A(\cdot )v||}_{C(E)}\leq M_{C}\left[{||f||}_{C(E)}+{||\varphi ||}_{D}\right], \end{array}$$

where *M*_*C*_  (1 ≤ *M*_*C*_ < +*∞*) does not depend on *φ*  and *f*(*t*).

Inequality (32) is called the coercivity inequality in *C*(*E*) for (27).  If *A*(*t*) = *A*, then the coercivity inequality implies the analyticity of the semigroup exp{−*sA*}  (*s* ≥ 0). Thus, the analyticity of the semigroup exp{−*sA*}  (*s* ≥ 0) is necessary for the well-posedness of problem (27) in *C*(*E*). Unfortunately, the analyticity of the semigroup exp{−*sA*}  (*s* ≥ 0) is not sufficient for the well-posedness of problem (27) in *C*(*E*).

A function *v*(*t*) is said to be a solution of problem (27) in *F*(*E*) if it is a solution of this problem in *C*(*E*) and the functions  *v*′(*t*) and *A*(*t*)*v*(*t*) belong to *F*(*E*).

As in the case of the space *C*(*E*), we say that the problem (27) is well posed in *F*(*E*) if the following two conditions are satisfied.

(1) For any *f* ∈ *F*(*E*) and *φ* ∈ *D*, there exists the unique solution *v*(*t*) = *v*(*t*; *f*(*t*), *φ*) in *F*(*E*) of problem (27). This means that an additive and homogeneous operator *v*(*t*; *f*(*t*), *φ*) is defined which acts from *F*(*E*) × *D* to *F*(*E*) and gives the solution of (27) in *F*(*E*).
(2) *v*(*t*; *f*(*t*), *φ*), regarded as an operator from *F*(*E*) × *D* to *F*(*E*), is continuous. Here, *F*(*E*) × *D* is understood as the normed space of the pairs (*f*(*t*), *φ*),*f*(*t*) ∈ *F*(*E*), and *φ* ∈ *D*, equipped with the norm
  (33) $$\begin{array}{c} {||\left(f(t),\varphi \right)||}_{F(E)\times D}={||f||}_{F(E)}+{||\varphi ||}_{D}. \end{array}$$

If *v*(*t*) is a solution in *C*_0_^*β*,*γ*^(*E*) of problem (27), then it is a solution in *C*(*E*) of this problem. Hence, by (13), we get the following representation for the solution of problem (27):

(34) v(t)=v(t,0)v(0)+∫0tv(t,s)f(s)ds,v(0)=(I−v(λ,0))−1(∫0λv(λ,s)f(s)ds+φ).

Using (27) and formula (34), we get

(35) v0′=v′(0)=−A(0)v(0)+f(0)=A(0)(I−v(λ,0))−1∫0λv(λ,s)(f(λ)−f(s))ds=A(0)(I−v(λ,0))−1∫0λv(λ,s)(f(λ)−f(s))ds +A(0)(I−v(λ,0))−1A−1(0)(−A(0)φ−f(λ)+f(0)) −A(0)(I−v(λ,0))−1 ×∫0λv(λ,s)[A(λ)−A(s)]A−1(λ)f(λ)ds +A(0)(I−v(λ,0))−1v(λ,0) ×(A−1(λ)f(λ)−A−1(0)f(0)) +A(0)(I−v(λ,0))−1v(λ,0)A−1(λ) ×(A(λ)−A(0))A−1(0)f(λ)=K1+K2+K3+K4,

where

(36) K1=A(0)(I−v(λ,0))−1×∫0λv(λ,s)(f(λ)−f(s))ds,K2=−A(0)(I−v(λ,0))−1×∫0λv(λ,s)[A(λ)−A(s)]A−1(λ)f(λ)ds,K3=A(0)(I−v(λ,0))−1A−1(0)×(−A(0)φ−f(λ)+f(0)),K4=A(0)(I−v(λ,0))−1v(λ,0)×(A−1(λ)f(λ)−A−1(0)f(0))+A(0)(I−v(λ,0))−1v(λ,0)A−1(λ)×(A(λ)−A(0))A−1(0)f(λ).

The main result of the present paper is the following theorem on well-posedness of (27) in the spaces *C*_0_^*β*,*γ*^(*E*_*α*−*β*_), 0 ≤ *γ* ≤ *β* ≤ *α*, 0 < *α* < 1.

**Theorem 9**

Suppose *f*(*t*) ∈ *C*_0_^*β*,*γ*^(*E*_*α*−*β*_)  (0 ≤ *γ* ≤ *β* ≤ *α*, 0 < *α* < 1). Suppose that the assumptions (9) and (10) hold and 0 < *α* ≤ *ε* < 1. If *A*(0)*φ* + *f*(*λ*) − *f*(0) ∈ *E*_*α*−*γ*_, then for the solution *v*(*t*) in *C*_0_^*β*,*γ*^(*E*_*α*−*β*_) of the nonlocal boundary value problem (27) the coercive inequality

(37) ||v′||C0β,γ(Eα−β)+||A(·)v||C0β,γ(Eα−β)+||v′||C(Eα−γ) ≤M(λ)α−1(1−α)−1  ×[||A(0)φ+f(λ)−f(0)||Eα−γ+||f||C0β,γ(Eα−β)]

holds, where *M*(*λ*) does not depend on *α*, *β*, *γ*, *φ*, and *f*(*t*).

**Proof**

The proof of Theorem 9 is based on Theorem 5 and the following estimate:

(38) ||v0′||Eα−γ≤M(λ)α−1(1−α)−1×[||A(0)φ+f(λ)−f(0)||Eα−γ+fC0β,γ(Eα−β)].

Let us estimate *K*_*m*_ for any *m* = 1,2, 3,4 in *E*_*α*−*γ*_, separately. We start with *K*_1_. Applying the inequality (30), we get

(39) $$\begin{array}{c} {||K_{1}||}_{E_{\alpha -\gamma }}\leq M\left(\lambda \right){||A\left(\lambda \right)\overset{\lambda }{\underset{0}{\int }}v\left(\lambda ,s\right)\left(f\left(\lambda \right)-f\left(s\right)\right)ds||}_{E_{\alpha -\gamma }}. \end{array}$$

Therefore, we will estimate

(40) $$\begin{array}{c} {||A\left(\lambda \right)\overset{\lambda }{\underset{0}{\int }}v\left(\lambda ,s\right)\left(f\left(\lambda \right)-f\left(s\right)\right)ds||}_{E_{\alpha -\gamma }}. \end{array}$$

We have that

(41) A(λ)∫0λv(λ,s)(f(λ)−f(s))ds =A(λ)∫0λexp{−(λ−s)A(s)}(f(λ)−f(s))ds  +A(λ)∫0λ[v(λ,s)−exp{−(λ−s)A(λ)}]    ×(f(λ)−f(s))ds =K11+K12,

where

(42) K11=A(λ)∫0λexp{−(λ−s)A(λ)}(f(λ)−f(s))ds,K12=A(λ)∫0λ[v(λ,s)−exp{−(λ−s)A(λ)}] ×(f(λ)−f(s))ds.

By the definition of spaces *E*_*α*−*β*_ and using estimates (9), we obtain

(43) z1−(α−γ)||A(λ)exp{−zA(λ)}K11||E≤z1−(α−γ)||A(λ)exp{−zA(λ)}×∫0λA(λ)exp{−(λ−s)A(λ)}(f(λ)−f(s))ds||E≤z1−α+γ∫0λ||A2(λ)exp{−(λ−s+z)A(λ)}f(λ)−f(s)||Eds≤Mz1−α+γ∫0λ(λ−s)βds(z+λ−s)2−α+βλγ||f||C0β,γ(Eα−β)

for any *z* > 0. We will consider separately the cases *z* ≤ *λ* and *z* > *λ*. If *z* ≤ *λ*, then

(44) z1−α+γ∫0λ(λ−s)βds(z+λ−s)2−α+βλγ ≤z1−α+γλγ∫0λds(z+λ−s)2−α ≤z1−α+γ(1−α)λγz1−α≤11−α(zλ)γ≤11−α.

If *z* > *λ*, then

(45) z1−α+γ∫0λ(λ−s)βds(z+λ−s)2−α+βλγ ≤1zα−γλγ∫0λds(z+λ−s)1−α ≤1λα−γλγ∫0λds(λ−s)1−α=1α.

Combining these estimates, we get

(46) $$\begin{array}{c} z^{1-(\alpha -\gamma )}{||A\left(\lambda \right)\exp\{-zA(\lambda )\}K_{11}||}_{E}\leq \frac{M}{\alpha \left(1-\alpha \right)}{||f||}_{C_{0}^{\beta ,\gamma }(E_{\alpha -\beta })} \end{array}$$

for any *z* > 0. Therefore,

(47) $$\begin{array}{c} {||K_{11}||}_{E_{\alpha -\gamma }}\leq \frac{M}{\alpha \left(1-\alpha \right)}{||f||}_{C_{0}^{\beta ,\gamma }(E_{\alpha -\beta })}. \end{array}$$

By the definition of spaces *E*_*α*−*β*_ and using estimates (9), (18), (19), and (28), we obtain

(48) z1−(α−γ)||A(λ)exp{−zA(λ)}K12||E ≤z1−(α−γ)||A(λ)exp{−zA(λ)}∫0λA(λ)×[v(λ,s)−exp{−(λ−s)A(λ)}]×(f(λ)−f(s))ds||E ≤z1−α+γ∫0λ||A2(λ)exp{−zA(λ)}  ×[v(λ,s)−exp{−(λ−s)A(λ)}]||E→E     ×||f(λ)−f(s)||Eα−βds ≤Mz1−α+γ∫0λmin[1z2,1(λ−s)2](λ−s)ε(λ−s)βλ−γds  ×||f||C0β,γ(Eα−β) ≤M1z1−α+γ∫0λ(λ−s)ε(λ−s)βds(z+λ−s)2λγ||f||C0β,γ(Eα−β)

for any *z* > 0. We will consider separately the cases *z* ≤ *λ* and *z* > *λ*. If *z* ≤ *λ*, then

(49) z1−α+γ∫0λ(λ−s)ε(λ−s)βds(z+λ−s)2λγ ≤z1−α∫0λ(λ−s)αds(z+λ−s)2λε−α+β ≤z1−α∫0λds(z+λ−s)2−αTε−α+β≤T21−α.

If *z* > *λ*, then

(50) z1−α+γ∫0λ(λ−s)ε(λ−s)βds(z+λ−s)2λγ ≤z1−α+γ∫0λ(λ−s)αds(z+λ−s)2λγλε−α+β ≤1zα−γλγ∫0λds(λ−s)1−αTε−α+β≤λα−γαzα−γT2<T2α.

Combining these estimates, we get

(51) $$\begin{array}{c} z^{1-(\alpha -\gamma )}{||A\left(\lambda \right)\exp\left\{-zA\left(\lambda \right)\right\}K_{12}||}_{E}\leq \frac{M}{\alpha \left(1-\alpha \right)}{||f||}_{C_{0}^{\beta ,\gamma }(E_{\alpha -\beta })} \end{array}$$

for any *z* > 0. Therefore,

(52) $$\begin{array}{c} {||K_{12}||}_{E_{\alpha -\gamma }}\leq \frac{M}{\alpha \left(1-\alpha \right)}{||f||}_{C_{0}^{\beta ,\gamma }(E_{\alpha -\beta })}. \end{array}$$

Applying the triangle inequality and estimates (47) and (52), we obtain

(53) ||A(λ)∫0λv(λ,s)(f(λ)−f(s))ds||Eα−γ ≤Mα(1−α)||f||C0β,γ(Eα−β).

From this estimate and estimate (39), it follows the following estimate

(54) $$\begin{array}{c} {||K_{1}||}_{E_{\alpha -\gamma }}\leq \frac{M}{\alpha \left(1-\alpha \right)}{||f||}_{C_{0}^{\beta ,\gamma }(E_{\alpha -\beta })}. \end{array}$$

Now, we estimate *K*_2_. Applying inequality (30), we get

(55) ||K2||Eα−γ≤M(λ)||A(λ)∫0λv(λ,s)×[A(λ)−A(s)]A−1(λ)f(λ)ds||Eα−γ.

Using estimates (9), (18), (19), and (28), we obtain

(56) z1−(α−γ)||A(λ)exp{−zA(λ)}∫0λA(λ)v(λ,s)×[A(λ)−A(s)]A−1(λ)f(λ)ds||E ≤z1−α+γ∫0λ||A2(λ)exp{−zA(λ)}v(λ,s)||E→E  ×||[A(λ)−A(s)]A−1(λ)||E→E||f(λ)||Eds ≤z1−α+γ∫0λmin[1z2,1(λ−s)2](λ−s)εds||f||C(E) ≤M1z1−α+γ∫0λ(λ−s)εds(z+λ−s)2||f||C0β,γ(Eα−β)

for all *z* > 0. We will prove that

(57) $$\begin{array}{c} z^{1-\alpha +\gamma }\overset{\lambda }{\underset{0}{\int }}\frac{{\left(\lambda -s\right)}^{\varepsilon }ds}{{\left(z+\lambda -s\right)}^{2}}\leq \frac{M}{\alpha \left(1-\alpha \right)} \end{array}$$

for any *z* > 0. If *z* ≤ *λ*, then

(58) z1−α+γ∫0λ(λ−s)εds(z+λ−s)2 ≤z1−αλγ+ε−α∫0λds(z+λ−s)2−α≤λγ+ε−α1−α≤T21−α.

If *λ* < *z*, then

(59) z1−α+γ∫0λ(λ−s)εds(z+λ−s)2≤1zα−γ∫0λds(λ−s)1−ε=λεεzα−γ<λεελα−γ≤T2α.

Combining these estimates, we get (57). Applying estimates (55), (56), and (57), we obtain

(60) $$\begin{array}{c} {||K_{2}||}_{E_{\alpha -\gamma }}\leq \frac{M}{\alpha \left(1-\alpha \right)}{||f||}_{C_{0}^{\beta ,\gamma }(E_{\alpha -\beta })}. \end{array}$$

Applying inequality (30), we get

(61) $$\begin{array}{c} {||K_{3}||}_{E_{\alpha -\gamma }}\leq M\left(\lambda \right){||A\left(0\right)\varphi +f(\lambda )-f\left(0\right)||}_{E_{\alpha -\gamma }}. \end{array}$$

Finally, we estimate *K*_4_. Applying inequality (30), we get

(62) ||K4||Eα−γ ≤M1(λ)||A(λ)v(λ,0)[A−1(λ)f(λ)−A−1(0)f(0) +A−1(λ)[A(λ)−A(0)]A−1(0)f(λ)]||Eα−γ.

Using estimates (9), (18), (19), and (28), we obtain

(63) z1−(α−γ)||A2(λ)exp{−zA(λ)}v(λ,0)   ×(A−1(λ)f(λ)−A−1(0)f(0)   +A−1(λ)(A(λ)−A(0))A−1(0)f(λ))||E ≤z1−α+γ||A2(λ)exp{−zA(λ)}v(λ,0)A−1(λ)||E→E  ×[||f(λ)||E+||A(λ)A−1(0)||E→E||f(0)||E    +||[A(λ)−A(0)]A−1(0)||E→E||f(λ)||E] ≤Mz1−α+γmin[1z,1λ]||f||C0β,γ(E) ≤M1z1−α+γz+λ||f||C0β,γ(E)≤M2λ−α+γ||f||C0β,γ(Eα−β)

for all *z* > 0. Applying estimates (63) and (62), we get

(64) $$\begin{array}{c} {||K_{4}||}_{E_{\alpha -\gamma }}\leq M_{2}\left(\lambda \right){||f||}_{C_{0}^{\beta ,\gamma }(E_{\alpha -\beta })}. \end{array}$$

Combining the estimates (54), (60), (61), and (64), we get estimate (38). Theorem 9 is proved.

Furthermore, the method of proof of Theorem 6 and scheme of proof of Theorem 9 enable us to establish the following theorem on well-posedness of (27) in spaces *C*_0_^*β*,*γ*^(*E*_*α*−*β*_),  0 ≤ *γ* ≤ *β* ≤ *α*,  0 < *α* < 1.

**Theorem 10**

Suppose *f*(*t*) ∈ *C*_0_^*β*,*γ*^(*E*_*α*−*β*_)  (0 ≤ *γ* ≤ *β* ≤ *α*, 0 < *α* < 1). Suppose that the assumptions (9) and (10) hold and 0 < *α* ≤ *ε* < 1. If *A*(0)*φ* + *f*(*λ*) − *f*(0) ∈ *E*_*α*−*β*_^*β*,*γ*^, then for the solution *v*(*t*) in *C*_0_^*β*,*γ*^(*E*_*α*−*β*_) of the nonlocal boundary value problem (27) the coercive inequality

(65) ||v′||C0β,γ(Eα−β)+||A(·)v||C0β,γ(Eα−β)+||v′||C(Eα−ββ,γ) ≤M(λ)[|A(0)φ+f(λ)−f(0)|α−ββ,γ+α−1(1−α)−1||f||C0β,γ(Eα−β)]

holds, where *M*(*λ*) does not depend on *α*, *β*, *γ*, *φ*, and *f*(*t*).

Note that the spaces of smooth functions *C*_0_^*β*,*γ*^(*E*_*α*−*β*_)  (0 ≤ *γ* ≤ *β* ≤ *α*, 0 < *α* < 1), in which coercive solvability has been established, depend on the parameters *α*, *β*, and *γ*. However, the constants in the coercive inequalities depend only on *α*. Hence, we can choose the parameters *β* and *γ* freely, which increases the number of functional spaces in which problem (27) is well posed. In particular, Theorems 9 and 10 imply theorems on well-posedness of the nonlocal boundary value problem (27) in *C*_0_^*β*,*γ*^(*E*)  (0 ≤ *γ* ≤ *β*, 0 < *β* < 1) which is established in the paper [41].

## 3. Applications

First, we consider the nonlocal boundary value problem for the parabolic equation

(66) ∂u∂t−a(t,x)∂2u∂x2+δu=f(t,x),  0<t<T, 0<x<1,u(0,x)=u(λ,x)+φ(x), 0≤x≤1,u(t,0)=u(t,1), ux(t,0)=ux(t,1), 0≤t≤T,

where *φ*(*x*)(*x* ∈ [0,1]),  *a*(*t*, *x*), and *f*(*t*, *x*)  (*t* ∈ (0, *T*), *x* ∈ (0,1)) are given sufficiently smooth functions and *a*(*t*, *x*) = *a*(*t* + *λ*, *x*) ≥ *a* > 0 and they satisfy every compatibility conditions which guarantee that the problem (66) has a smooth solution *u*(*t*, *x*). Here,  *δ* > 0 is a sufficiently large number.

We introduce the Banach spaces *C* ^*β*^[0,1]  (0 < *β* < 1) of all continuous functions *φ*(*x*) satisfying a Hölder condition for which the following norms are finite:

(67) $$\begin{array}{c} {||\varphi ||}_{C^{\beta }[0,1]}={||\varphi ||}_{C[0,1]}+\underset{0\leq x<x+\tau \leq 1}{\sup}\frac{\left|\varphi \left(x+\tau \right)-\varphi \left(x\right)\right|}{\tau^{\beta }}, \end{array}$$

where *C*[0,1] is the space of the all continuous functions *φ*(*x*) defined on [0,1] with the usual norm

(68) $$\begin{array}{c} {||\varphi ||}_{C[0,1]}=\underset{0\leq x\leq 1}{\max}\left|\varphi \left(x\right)\right|. \end{array}$$

It is known that the differential expression

(69) $$\begin{array}{c} A^{t,x}v\left(t,x\right)=-a\left(t,x\right)v_{xx}\left(t,x\right)+\delta v\left(t,x\right) \end{array}$$

defines a strongly positive operator *A* ^*t*,*x*^ acting in *C* ^*β*^[0,1] with the domain *C* ^*β*+2^[0,1] and satisfying nonlocal boundary conditions *v*(*t*, 0) = *v*(*t*, 1), *v*_*x*_(*t*, 0) = *v*_*x*_(*t*, 1) for any 0 ≤ *t* ≤ *T*.

Therefore, we can replace the nonlocal boundary value problem (66) by the abstract nonlocal boundary value problem (27) in a Banach space *E* = *C*[0,1] with a strongly positive operator *A* ^*t*,*x*^ defined by formula (69).

**Theorem 11**

For the solution of nonlocal boundary value problem (66), the following coercive inequality:

(70) ||u||C01+β,γ(C2(α−β)+μ[0,1])+||u||C0β,γ(C2(α−β)+μ[0,1])  +||u||C(C2(α−γ)+μ[0,1]) ≤M(λ,μ,α)[||f||C0β,γ(C2(α−β)+μ[0,1])    +||−a(0,·)∂2φ(·)∂x2+δφ(·)   +f(λ,·)−f(0,·)||C2(α−γ)+μ[0,1]],0<2(α−γ)+μ<1,0≤γ≤β, 0≤μ≤1, 0<α<1

holds, where *M*(*λ*, *μ*, *α*) does not depend on *γ*, *β*,  *f*(*t*, *x*),  *φ*(*x*).

The proof of Theorem 11 is based on abstract Theorem 9 and on the following theorem on the structure of fractional spaces *E*_*α*_(*C* ^*μ*^[0,1], *A* ^*t*,*x*^).

**Theorem 12**

*E*_*α*_(*C* ^*μ*^[0,1], *A* ^*t*,*x*^) = *C* ^2*α*+*μ*^[0,1] for all 0 < 2*α* + *μ* < 1,  0 ≤ *μ* ≤ 1 and 0 ≤ *x* ≤ 1,  0 ≤ *t* ≤ *T* [42].

Second, on the range {0 ≤ *t* ≤ *T*, *x* ∈ ℝ^*n*^}, we consider the nonlocal boundary value problem for the 2*m*th order multidimensional parabolic equation

(71) $$\begin{array}{c} \frac{\partial u}{\partial t}+\underset{\left|r\right|=2m}{\sum }a_{r}\left(t,x\right)\frac{\partial^{\left|\tau \right|}u}{\partial x_{1}^{r_{1}},\ldots ,\partial x_{n}^{r_{n}}}+\delta u\left(t,x\right)=f\left(t,x\right),\\ 0<t<T,\;x,r\in {\mathbb{R}}^{n},\;\left|r\right|=r_{1}+\cdots +r_{n},\\ u\left(0,x\right)=u\left(\lambda ,x\right)+\varphi \left(x\right),\;x\in {\mathbb{R}}^{n}, \end{array}$$

where *a*_*r*_(*t*, *x*) = *a*_*r*_(*t* + *λ*, *x*)  and *φ*(*x*)(*x* ∈ ℝ^*n*^),  *a*_*r*_(*t*, *x*), and *f*(*t*, *x*)  (*t* ∈ (0, *T*), *x* ∈ ℝ^*n*^) are given sufficiently smooth functions. Here, *δ* > 0 is a sufficiently large number.

Let us consider a differential operator with constant coefficients of the form

(72) $$\begin{array}{c} B=\underset{\left|r\right|=2m}{\sum }b_{r}\frac{\partial^{r_{1}+\cdots +r_{n}}}{\partial x_{1}^{r_{1}},\ldots ,\partial x_{n}^{r_{n}}}, \end{array}$$

acting on functions defined on the entire space  *R* ^*n*^. Here, *r* ∈ *R* ^*n*^ is a vector with nonnegative integer components,  |*r*| = *r*_1_ + ⋯+*r*_*n*_. If *φ*(*y*)  (*y* = (*y*_1_,…, *y*_*n*_) ∈ *R* ^*n*^) is an infinitely differentiable function that decays at infinity together with all its derivatives, then, by means of the Fourier transformation one establishes the equality

(73) $$\begin{array}{c} F\left(B_{\varphi }\right)\left(\xi \right)=B\left(\xi \right)F\left(\varphi \right)\left(\xi \right). \end{array}$$

Here, the Fourier transform operator is defined by the rule

(74) F(φ)(ξ)=(2π)−n/2∫Rnexp{−i(y,ξ)}φ(y)dy,(y,ξ)=y1ξ1+⋯+ynξn.

The function *B*(*ξ*) is called the symbol of the operator *B* and is given by  

(75) $$\begin{array}{c} B\left(\xi \right)=\underset{\left|r\right|=2m}{\sum }b_{r}{\left(i\xi_{1}\right)}^{r_{1}},\ldots ,{\left(i\xi_{n}\right)}^{r_{n}}. \end{array}$$

We will assume that the symbol

(76) Bt,x(ξ)=∑|r|=2mar(t,x)(iξ1)r1,…,(iξn)rn,ξ=(ξ1,…,ξn)∈Rn

of the differential operator of the form

(77) $$\begin{array}{c} B^{t,x}=\underset{\left|r\right|=2m}{\sum }a_{r}\left(t,x\right)\frac{\partial^{\left|r\right|}}{\partial x_{1}^{r_{1}},\ldots ,\partial x_{n}^{r_{n}}}, \end{array}$$

acting on functions defined on the space ℝ^*n*^, satisfies the inequalities

(78) $$\begin{array}{c} 0<M_{1}{\left|\xi \right|}^{2m}\leq {\left(-1\right)}^{m}B^{t,x}\left(\xi \right)\leq M_{2}{\left|\xi \right|}^{2m}<\infty \end{array}$$

for *ξ* ≠ 0 and for any 0 ≤ *t* ≤ *T*. Assume that the all compatibility conditions hold which guarantees that the problem (71) has a unique smooth solution *u*(*t*, *x*). This allows us to reduce the nonlocal boundary value problem (71) by the abstract nonlocal boundary value problem (27) in a Banach space *E* = *C* ^*μ*^(*R* ^*n*^) of all continuous bounded functions defined on ℝ^*n*^ satisfying a Hölder condition with the indicator *μ* ∈ (0,1) with a strongly positive operator *A* ^*t*,*x*^ = *B* ^*t*,*x*^ + *δI* defined by (77).

**Theorem 13**

For the solution of the nonlocal boundary value problem (71), the following coercivity inequality:

(79) ||u||C01+β,γ(C2m(α−β)+μ(Rn)) +∑|τ|=2m||∂|r|u∂x1r1,…,∂xnrn||C0β,γ(C2m(α−β)+μ(Rn)) +||u||C(C2m(α−γ)+μ(Rn))≤M(λ,μ,α)[||f||C0β,γ(C2m(α−β)+μ(Rn))+||∑|r|=2mar(0,·)∂|τ|φ(·)∂x1r1,…,∂xnrn+δφ(·)      +f(λ,·)−f(0,·)||C2m(α−γ)+μ(Rn)],0<2(α−γ)+μ<1, 0≤γ≤β, 0≤μ≤1

holds, where *M*(*λ*, *μ*, *α*) does not depend on  *γ*,  *β*,  *f*(*t*, *x*), *φ*(*x*).

The proof of Theorem 13 is based on abstract Theorem 9, on the coercivity inequality for an elliptic operator *A* ^*t*,*x*^ in *C* ^*μ*^(*R* ^*n*^), and on the following theorem on the structure of fractional spaces *E*_*α*_(*C* ^*μ*^(*R* ^*n*^), *A* ^*t*,*x*^).

**Theorem 14**

*E*_*α*_(*C* ^*μ*^(*R* ^*n*^), *A* ^*t*,*x*^) = *C* ^2*mα*+*μ*^(*R* ^*n*^) for all 0 < 2*mα* + *μ* < 1,  0 ≤ *μ* ≤ 1  and *x* ∈ ℝ^*n*^,  0 ≤ *t* ≤ *T* [7].

## 4. Conclusion

In the present study, the well-posedness of the nonlocal boundary value parabolic problem with dependent coefficients in Hölder spaces with a weight is established. In practice, new Schauder type exact estimates in Hölder norms for the solution of two nonlocal boundary value problems for parabolic equations with dependent coefficients are obtained. Moreover, applying the result of the monograph [14], the high order of accuracy single-step difference schemes for the numerical solution of the nonlocal boundary value parabolic problem with dependent coefficients can be presented. Of course, the coercive stability estimates for the solution of these difference schemes have been established without any assumptions about the grid steps.
